# The influence of μ-opioid and noradrenaline reuptake inhibition in the modulation of pain responsive neurones in the central amygdala by tapentadol in rats with neuropathy

**DOI:** 10.1016/j.ejphar.2014.11.032

**Published:** 2015-02-15

**Authors:** Leonor Gonçalves, Lauren V. Friend, Anthony H. Dickenson

**Affiliations:** Neuroscience Physiology & Pharmacology, Medical Sciences Building, University College London, Gower St, London WC1E 6BT, UK

**Keywords:** Rat, Tapentadol, Neuropathy, Right central amygdala, Opioid, Noradrenaline

## Abstract

Treatments for neuropathic pain are either not fully effective or have problematic side effects. Combinations of drugs are often used. Tapentadol is a newer molecule that produces analgesia in various pain models through two inhibitory mechanisms, namely central μ-opioid receptor (MOR) agonism and noradrenaline reuptake inhibition. These two components interact synergistically, resulting in levels of analgesia similar to opioid analgesics such as oxycodone and morphine, but with more tolerable side effects. The right central nucleus of the amygdala (CeA) is critical for the lateral spinal ascending pain pathway, regulates descending pain pathways and is key in the emotional-affective components of pain. Few studies have investigated the pharmacology of limbic brain areas in pain models. Here we determined the actions of systemic tapentadol on right CeA neurones of animals with neuropathy and which component of tapentadol contributes to its effect. Neuronal responses to multimodal peripheral stimulation of animals with spinal nerve ligation or sham surgery were recorded before and after two doses of tapentadol. After the higher dose of tapentadol either naloxone or yohimbine were administered. Systemic tapentadol resulted in dose-dependent decrease in right CeA neuronal activity only in neuropathy. Both naloxone and yohimbine reversed this effect to an extent that was modality selective.

The interactions of the components of tapentadol are not limited to the synergy between the MOR and α_2_-adrenoceptors seen at spinal levels, but are seen at this supraspinal site where suppression of responses may relate to the ability of the drug to alter affective components of pain.

## 1 Introduction

Approximately 8% of the world population is affected by neuropathic pain (Tzschentke et al., 2007). Treatment options include anti-convulsants and antidepressants as well as opioids but overall, are either not very effective (Fishbain, 2000), or the common side effects are not well tolerated by patients (Katz and Benoit, 2005). Opioids, for example, provide a good level of analgesia, but besides the potential abuse (Kalso et al., 2004) and opioid-induced hyperalgesia (Bannister and Dickenson, 2010), side effects (constipation, nausea, sedation, respiratory depression) are common and impairing (Bruehl et al., 2013). Often, combinations of drugs are used to increase analgesia or reduce side-effects (Gilron et al., 2009).

Tapentadol is a recent drug with a dual mode of action that has been shown to produce an analgesic effect in chronic pain in humans (Hartrick and Rozek, 2011) and animals (Tzschentke et al., 2007), acting centrally through two mechanisms: μ-opioid receptor (MOR) agonism and noradrenaline reuptake inhibition (NRI), with the latter leading to α_2_-adrenoceptor activation (Tzschentke et al., 2007). Although each of the two components can produce analgesia independently, their synergistic interaction allows a moderate action of each, resulting in levels of analgesia similar to that of opioid analgesics such as oxycodone and morphine, but with reduced opioid load and so a more tolerable side effect profile (Riemsma et al., 2011; Schröder et al., 2011). Tapentadol action results, at least in part, from its interaction with spinal MOR and enhancement of spinal noradrenaline levels to activate α_2_-adrenoceptor inhibitions (Bee et al., 2011).

Relatively few analgesics have been studied on supraspinal structures and little is known about actions on areas involved in affective components of pain. This is important since patients report not only pain but reduced quality of life (ref). Supraspinally, the amygdala (AMY), mainly the right CeA (Carrasquillo and Gereau, 2008) is critically involved in the lateral spinal ascending pathways and in regulation of descending pain pathways. The AMY is key in the emotional-affective component of pain (Neugebauer et al., 2004; Neugebauer et al., 2009) and the central nucleus of the amygdala (CeA) has a foremost role, with a high number of nociceptive neurones (Neugebauer and Li, 2002) and strong responses to peripheral stimuli (Neugebauer et al., 2004). Activation of AMY neurones happens mostly after aversive stimuli through multiple sensory modalities (Fischer et al., 2003; Garrett and Maddock, 2001; Phan et al., 2004; Phelps et al., 2001; Zald and Pardo, 2002) and includes ongoing and evoked abnormal activity after neuropathy that dominates in the right CeA (Gonçalves and Dickenson, 2012). In this latter study, pregabalin was able to reduce neuronal activity in neuropathic animals although the ability of the drug to act as an anxiolytic as well as an analgesic complicates its effects.

In this study we aimed to determine if systemic tapentadol has an effect on the responsivity of right CeA neurones of animals with neuropathy and to clarify which component of this drug contributes to its effect. In order to achieve this we recorded and analyzed the activity of right CeA neurones of animals subjected to spinal nerve ligation (SNL) or sham surgery, before and after two doses of tapentadol. In addition, after the higher dose of tapentadol we administered either the MOR antagonist naloxone or the α_2-_adrenoceptor antagonist yohimbine.

## 2 Material and methods

All experimental procedures used in this study were approved by the UK Home Office and followed the guidelines under the International Association for the Study of Pain (Zimmermann, 1983). The electrophysiological data that resulted from these experiments were obtained from naïve animals and animals subjected to either sham or spinal nerve ligation surgery (SNL; Kim and Chung, 1992).

The numbers of animals used on this study were as follows: naïve *n*=8, sham *n*=22, SNL (tapentadol) *n*=21 and SNL (naloxone alone) *n*=4. From the 22 sham rats, 5 were recorded for extra 20 min after 5 mg/kg tapentadol with no further treatment, 7 were injected with naloxone and 10 injected with yohimbine. From the 21 SNL rats, 5 were recorded for extra 20 min after 5 mg/kg tapentadol with no further treatment, 8 were injected with naloxone and 8 injected with yohimbine.

### 2.1 Spinal nerve ligation

The spinal nerve ligation model (SNL) was induced through surgery and performed, as first described by Kim & Chung (1992), in the lower left lumbar spinal region of Sprague–Dawley male rats (Central Biological Services, University College London, London UK), initially weighing 130–150 g. Briefly, selective tight ligation of spinal nerves L5 and L6 is performed with 6–0 silk thread under isofluroane (1.5–1.7%) delivered in a gaseous mix of N_2_O (66%) and O_2_ (33%). The sham procedure is identical to similar to the normal SNL surgery, but omits the ligation of the nerves.

### 2.2 Electrophysiology

**Fig. 1:**
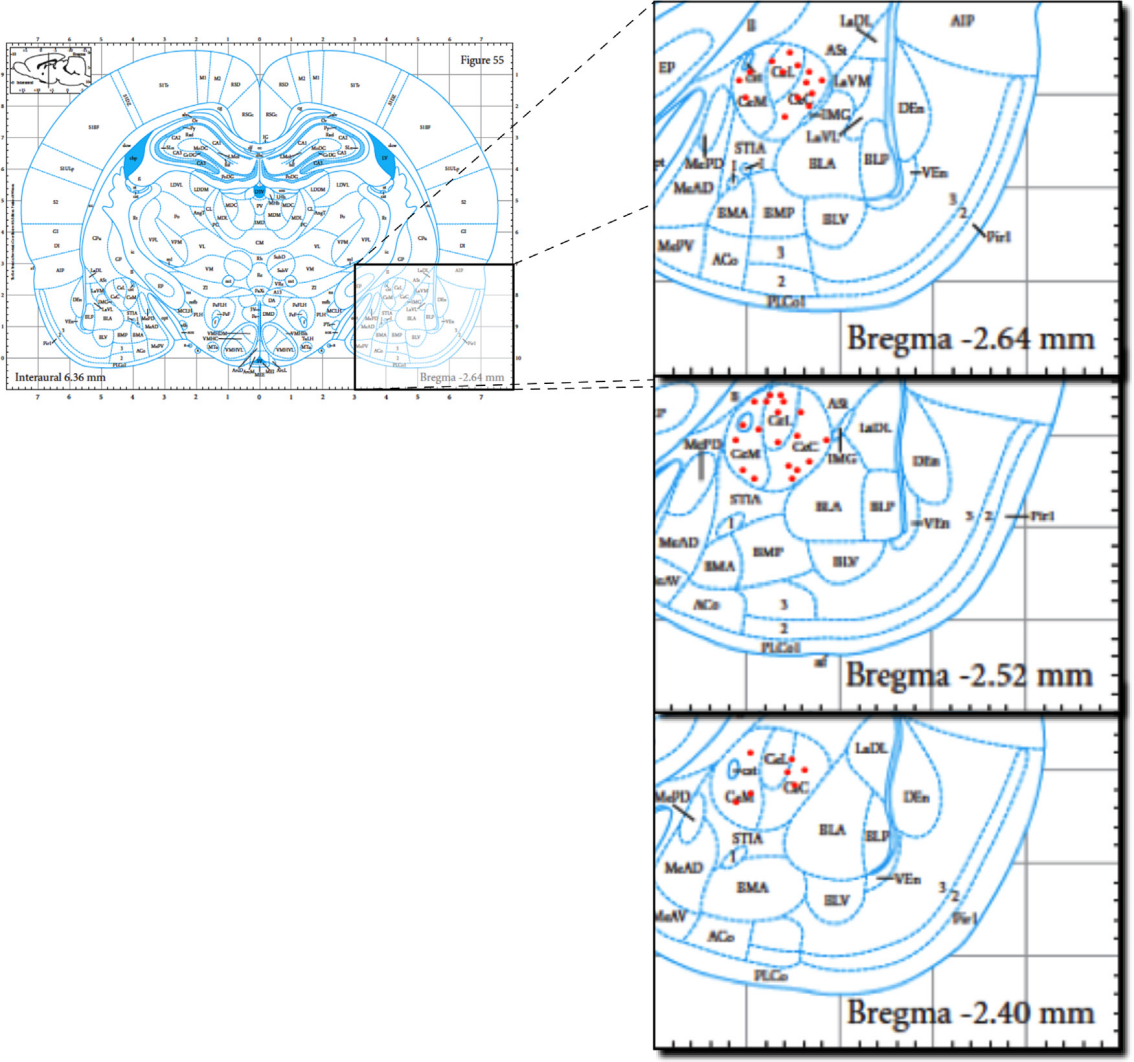
Sections of the atlas representing the placement of the recordings. The recordings (red dots) were located between 2.40 mm and 2.64 mm caudal, 7.40 mm and 8.50 mm ventral and 3.6 mm and 5 mm lateral (to the right) from bregma. (For interpretation of the references to color in this figure legend, the reader is referred to the web version of this article.)

Since in a previous study we showed that CeA neuronal evoked activity is increased and stable at 14 days after SNL surgery (Gonçalves and Dickenson, 2012), single neurones in the CeA were recorded extracellularly with parylene coated tungsten electrodes (A-M Systems, USA) on post-operative days 14–17, using the stereotaxic coordinates (Ji and Neugebauer, 2009): 1.6–3.2 mm caudal, 3.8–4.4 mm lateral and 7.8–8.6 mm ventral to bregma (Fig. 1). Only neurones from the right CeA were recorded in this study, given that previous studies showed that pain processing seems to be lateralized to right the AMY, and right CeA neurones have higher activity than left CeA neurones in inflammatory, arthritic and neuropathic pain (Carrasquillo and Gereau, 2008; Ji and Neugebauer, 2009; Gonçalves and Dickenson, 2012).

The animals were first placed in a induction box and anesthetized with isofluorane (5%) delivered in a gaseous mix of N2O (66%) and O2 (33%), were then transferred to a nose cone (receiving isofluorane (3%) delivered in the same gaseous mix) in order to perform a tracheotomy, after which they received the isofluorane (1.5–1.7%) and gaseous mix of N_2_O and O_2_ directly to the trachea, through a tube connected to the anesthetic and gases system. The animals were then maintained at this level of anesthesia with areflexia for the entire duration of the experiment.

Afterwards, the animals were fixed in a stereotaxic device and, after the skull was exposed, CeA coordinates were calculated after bregma (Ji and Neugebauer, 2009). Finally, a small craniotomy was performed with a drill and the dura matter removed, exposing the brain. To be considered for the experiment, neurones had to fire spontaneously and constantly for at least 20 min, after which two rounds of spontaneous and evoked activity were recorded (the average of these two rounds was considered to be baseline activity).

On SNL and sham animals, spontaneous and stimuli-activity of right CeA neurones (one per animal) were recorded and analyzed before (baseline) and 10, 20, 40 and 60 min after each dose of tapentadol, and 10 and 20 min after naloxone or yohimbine injection. In a separated set of naïve and SNL animals, the same recordings were made before and 10, 20, 40 and 60 min after naloxone administration. The following stimuli were applied for a period of 10 s (separated by a 40 s interval; Goncalves and Dickenson, 2012): von Frey filaments (0.008 g, 4.0 g, both innocuous and 60 g, noxious), cold (4 °C, acetone test) and heat (48 °C, constant water jet) applied on both hindpaws, and controlled pinch of right hindpaws, tail and right ear.

Data are captured by a CED 1401 interface coupled to a Pentium computer with Spike 2 software (Cambridge Electronic Design; PSTH and rate functions) and posteriorly analyzed.

### 2.3 Drug administration

In this study three different drugs were used: tapentadol HCl (MOR agonist and noradrenaline reuptake inhibitior; Grunenthal GmbH, Aachen, Germany), naloxone HCl (MOR antagonist; Sigma Chemie, Deisenhofen, Germany) and yohimbine HCl (α_2_-adrenoceptor antagonist, Sigma Chemie, Deisenhofen, Germany).

An injection of tapentadol 1 mg/kg was first administered via subcutaneous injection in the scruff of the back of the neck of each animal and activity of one spontaneously firing neurone is followed for over 60 min, with tests carried out at 10, 20, 40 and 60 min. 70 min after the first dose, a second (higher) dose of tapentadol (5 mg/kg) is administered in the same manner, and tests carried out at the same time points. One 70 min after the higher dose of tapentadol either the recordings are prolonged for an extra 20 min, with tests performed at 10 and 20 min (control for the maintenance of the effect of the high dose of tapentadol for a longer period), or one antagonist (naloxone 5 mg/kg or yohimbine 5 mg/kg) per animal is injected subcutaneously, and tests carried out after 10 and 20 min. Drug does were based on those previously shown to be effective at spinal levels (Bee et al., 2011).

### 2.4 Statistical analysis

Unless stated otherwise, all comparisons were analyzed through repeated measures ANOVA test and Bonferroni Post Hoc using IBM SPSS Statistics software (version 21.0.0.0).

## 3 Results

### 3.1 Spontaneous and evoked neuronal activity in the CeA of SNL rats is significantly higher than in sham rats

Right CeA neuronal activity in SNL rats is significantly higher than in sham rats in both spontaneous and evoked conditions (data not shown). As previously demonstrated (Gonçalves and Dickenson, 2012), baseline spontaneous activity in the right CeA neurones was 6.82–11.13 Hz for SNL rats but only 1.52–3.43 Hz for sham rats. Neuronal responses to different stimuli were also different, being greater after nerve injury: 6.7–11.69 Hz for SNL and 3.01–4.58 Hz for sham animals after 0.008 g von Frey; 8.59–11.16 Hz for SNL and 2.16–4.83 Hz for sham animals after 4 g von Frey; 8.48–11.85 Hz for SNL and 2.21–4.28 Hz for sham animals after 60 g von Frey; 10.39–14.47 Hz for SNL and 3.04–4.60 Hz for sham animals after cold; 11.48–14.10 Hz for SNL and 3.18–4.55 Hz for sham animals after heat; 10.93–15.245 Hz for SNL and 3.07–4.40 Hz for sham animals after paw pinch; 12.34–15.25 Hz for SNL and 2.86–3.94 Hz for sham animals after tail pinch; and 11.12–14.16 Hz for SNL and 2.70–4.72 Hz for sham animals after ear pinch.

### 3.2 Evoked neuronal activity in the right CeA is significantly higher than spontaneous activity in SNL animals

**Fig. 2:**
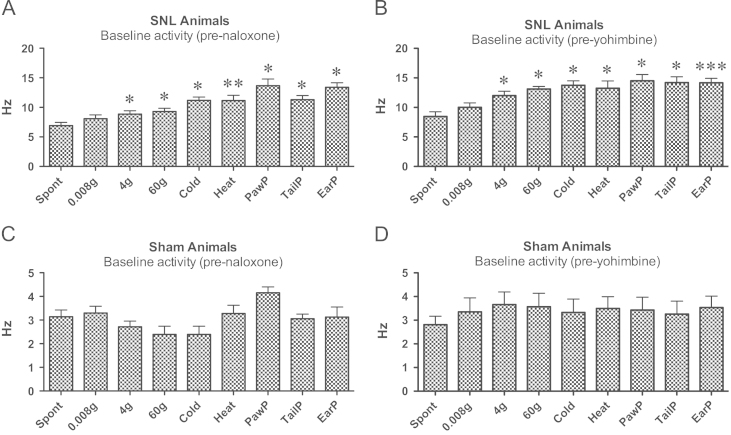
Comparison between spontaneous and evoked activities at baseline. SNL animals subsequently injected with tapentadol and naloxone (A) or yohimbine (B) showed significant differences between spontaneous activity and reponses to 4 g and 60 vonFrey, cold, heat, paw, tail and ear pinch. In sham animals there were no differences between spontaneous and evoked activity (C and D) ^⁎, ⁎⁎, ⁎⁎⁎^*P*<0.05, 0.01 and 0.001 for significant difference between spontaneous and evoked activity. Mean±S.E.M.

Responses evoked by all stimuli but 0.008 g von Frey were significantly higher than spontaneous activity recorded on the CeA of SNL animals during baseline (Fig. 2 A and B). Statistically analysis showed differences both in the group that subsequently received naloxone (Fig. 2 A; 4 g von Frey-evoked activity *P*=0.049, 60 g von Frey-evoked activity *P*=0.048; cold-evoked activity *P*=0.042, heat-evoked activity *P*=0.008, paw pinch-evoked activity *P*=0.020, tail pinch-evoked activity *P*=0.043, ear pinch-evoked activity *P*=0.019) and the group that subsequently received yohimbine (Fig. 2B; 4 g von Frey-evoked activity *P*=0.047, 60 g von Frey-evoked activity *P*=0.013; cold-evoked activity *P*=0.048, heat-evoked activity *P*=0.034, paw pinch-evoked activity *P*=0.014, tail pinch-evoked activity *P*=0.019, ear pinch-evoked activity *P*<0.001).

These changes were produced by the neuropathy as there were no statistically significant differences between spontaneous and evoked activity in right CeA neurones of sham animals (Fig. 2 C and D). For this reason, all the subsequent analysis were performed only on spontaneous activity values for this group.

### 3.3 Tapentadol and antagonists have different effects on spontaneous activity of CeA neurones in SNL and sham animals

#### 3.3.1 Effect of two doses of tapentadol and subsequent administration of antagonist on SNL animals

**Fig. 3:**
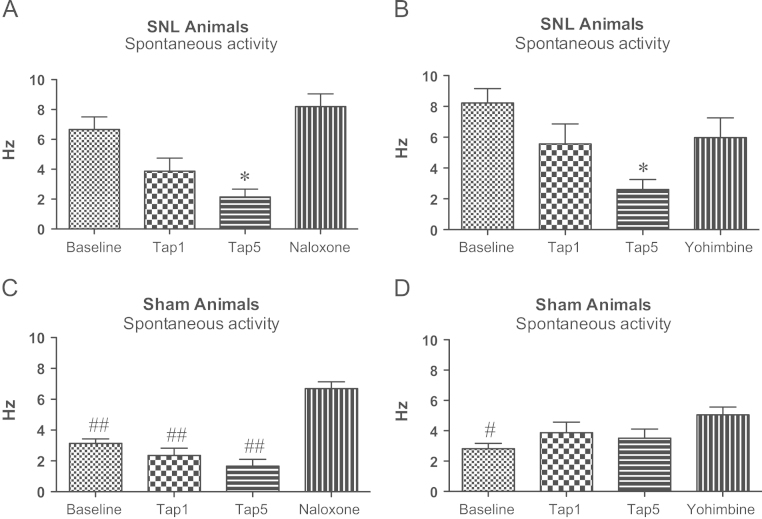
Effect of 1 and 5 mg/kg tapentadol and subsequent naloxone or yohimbine on spontaneous activity of SNL and sham animals. (A) 5 mg/kg tapentadol administration reduces SNL spontaneous activity, which is then reversed by 5 mg/kg naloxone. (B) 5 mg/kg tapentadol administration reduces SNL spontaneous activity, which is then reversed by 5 mg/kg yohimbine. (C, D) Tapentadol did not have a significant effect on spontaneous activity of sham animals. However, naloxone resulted in a significant increase of activity (C), as did yohimbine, although to a lesser extent (D). ^⁎^*P*<0.05 for significant difference between baseline and post-tapentadol administration. ^#, ##^*P*<0.05, 0.01 for significant difference between activity before and after naloxone or yohimbine. Mean±S.E.M.

Systemic administration of 1 mg/kg tapentadol results in an inhibition of neuronal activity of CeA neurones only in SNL animals that did not reach significance, while 5 mg/kg tapentadol result in a greater and enduring inhibition of the same activity (Fig. 3 A and B). In both groups the higher dose of tapentadol significantly reduced neuronal activity (RM ANOVA with Bonferroni pairwise comparisons): *P*=0.045 for naloxone group (Fig. 3 A) and *P*=0.032 for the yohimbine group (Fig. 3B). Both antagonists completely reversed the effect of tapentadol, as shown by the lack of difference between baseline activity and activity recorded after either naloxone (*P*=0.931) or yohimbine (*P*=1.000).

Thus, tapentadol significantly inhibited and spontaneous activities of these neurones and both antagonists reversed these effects, in that responses post-naloxone or post-yohimbine were no longer significantly different from the pre-tapentadol control values.

#### 3.3.2 Effect of two doses of tapentadol and subsequent administration of antagonist on sham animals

Tapentadol did not have a significant effect on the spontaneous activity of CeA neurones in sham animals (Fig. 3 C and D). However, naloxone (Fig. 3 C) resulted in a significant increase of activity relative to baseline (*P*=0.005), 1 mg/kg (*P*=0.001) and both 5 mg/kg tapentadol (*P*=0.001) and yohimbine (Fig. 3D) resulted in a significantly higher activity relatively to baseline (*P*=0.016), suggesting ongoing opioid and noradrenergic tone.

#### 3.3.3 Comparison between sham and SNL neuronal activity at baseline and after naloxone or yohimbine (post-tapentadol)

**Fig. 4:**
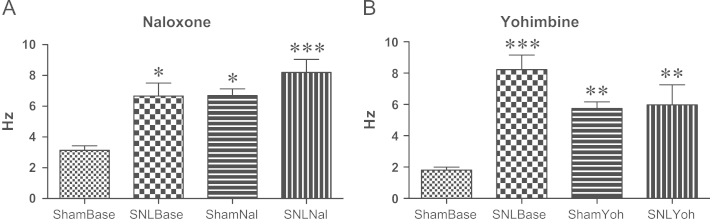
Comparison between SNL and sham CeA neuronal spontaneous activities at baseline and after naloxone or yohimbine. SNL and sham animals given tapentadol and naloxone (A) or yohimbine (B) showed significantly higher activity than spontaneous neuronal activity of sham animals at baseline. Spontaneous activity of neurones in the CeA of SNL animals at baseline was also given significantly higher activity than spontaneous neuronal activity of sham animals at baseline (A, B). ^⁎, ⁎⁎, ⁎⁎⁎^*P*<0.05, 0.01 and 0.001 for significant difference between sham baseline activity and any other of the 3 groups. Mean±S.E.M.

Baseline spontaneous activity of CeA neurones of SNL animals is significantly higher than the same activity in sham animals (*P*=0.016, Fig. 4 A; *P*<0.001, Fig. 4B). After naloxone (Fig. 4 A) or yohimbine (Fig. 4B), spontaneous activity of both SNL and sham animals was also significantly higher than baseline spontaneous activity of sham animals (SNL *P*<0.001 and sham *P*=0.019 after naloxone; both SNL and sham *P*=0.003 after yohimbine), but not different from the same activity in SNL animals. Comparisons of these data were analyzed through Two-Way ANOVA tests and Bonferroni Post Hoc using IBM SPSS Statistics software (version 21.0.0.0).

### 3.4 Evoked activity of CeA neurones in SNL animals is affected by tapentadol and antagonists

All values presented here and in the related graphs (Figs. 3 and 4) refer to the evoked responses minus its respective spontaneous activity. Since there was no significant difference between spontaneous activity and responses to the lowest mechanical stimulus, 0.008 g von Frey (Fig. 2A and B; see above), the results from the latter are not shown.

**Fig. 5:**
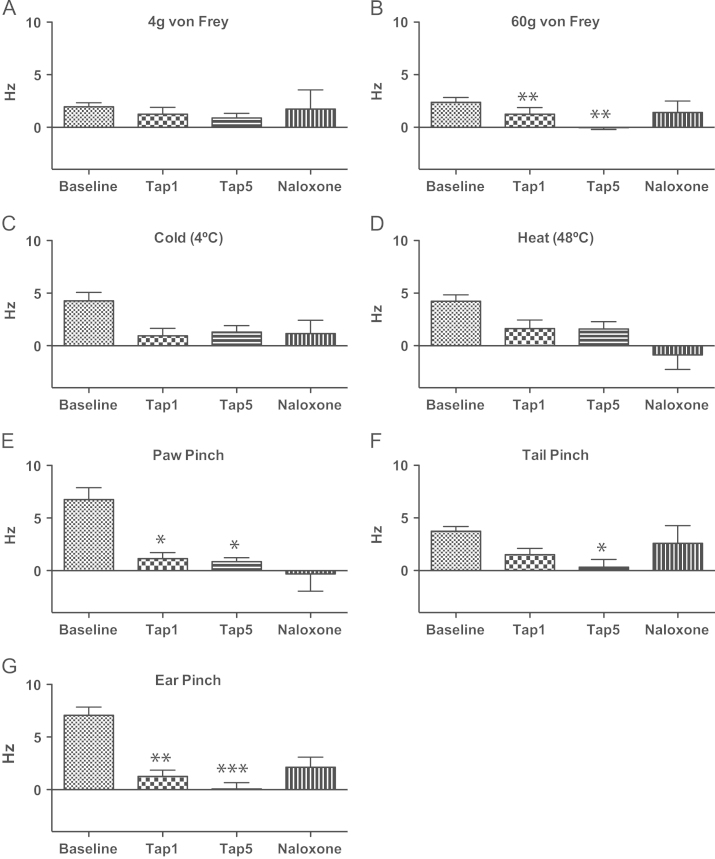
Effect of 1 and 5 mg/kg tapentadol and subsequent naloxone administration on evoked activity of SNL animals. Spontaneous activity was subtracted from the corresponding total activity after stimulation in order to obtain the real responses to stimuli. 1 and 5 mg/kg tapentadol and naloxone have no significant effect on the responses to 4g vonFrey (A), cold (C) and heat (D) stimulation. Responses to 60 g von Frey (B), paw pinch (E), and ear pinch (G) were significantly reduced by 1 and 5 mg/kg tapentadol and this effect was reversed by naloxone. Responses to tail pinch (F) were significantly reduced by 5 mg/kg tapentadol and this effect was reversed by naloxone. ^⁎, ⁎⁎, ⁎⁎⁎,^*P*<0.05, 0.01 and 0.001 for significant difference between baseline and post-tapentadol administration. Mean±S.E.M.

**Fig. 6:**
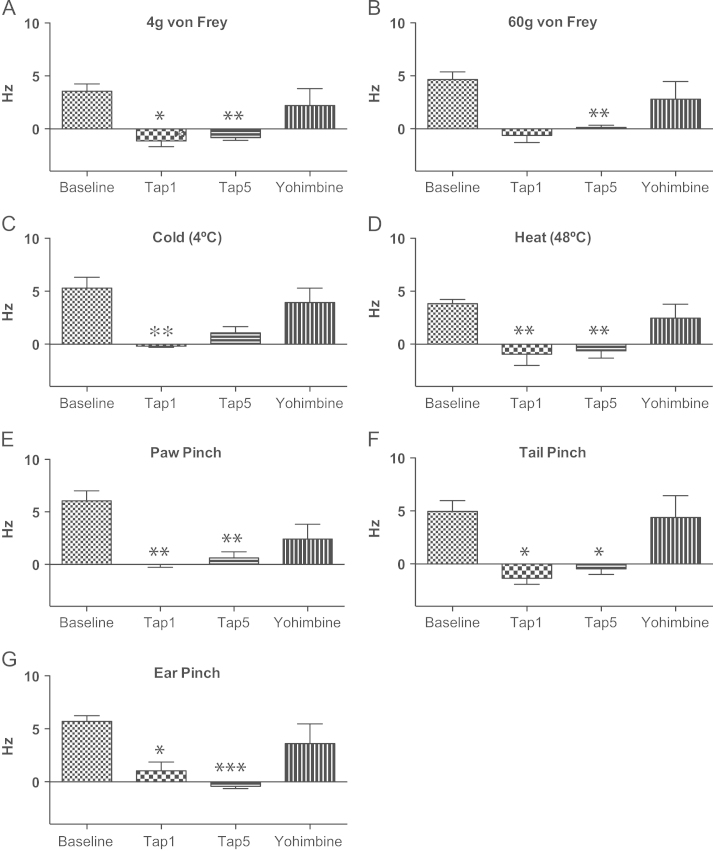
Effect of 1 and 5 mg/kg tapentadol and subsequent yohimbine administration on evoked activity of SNL animals. Spontaneous activity was subtracted from the corresponding total activity after stimulation in order to obtain the real responses to stimuli. Responses to 4 g vonFrey (A), heat (D), tail (F) and ear (G) pinch were reduced by 1 and 5 mg/kg tapentadol and this effect was completely reversed by yohimbine. (B) Responses to 60 g von Frey were significantly reduced by 5 mg/kg tapentadol and this effect was completely reversed by yohimbine. (C) Responses to cold were significantly reduced by 1 mg/kg tapentadol and this effect was almost completely reversed by yohimbine. (E) Responses to paw pinch were significantly reduced by 1 and 5 mg/kg tapentadol and this effect was partially reversed by yohimbine. ^⁎, ⁎⁎, ⁎⁎⁎^*P*<0.05, 0.01 and 0.001 for significant difference between baseline and post-tapentadol administration. Mean±S.E.M.

Neuronal CeA activity before and after tapentadol is presented separately for each of the groups of SNL animals that received either naloxone (Fig. 5) or yohimbine (Fig. 6). This resulted in different statistical results for each of the groups, with both groups showing a clear effect of tapentadol on spontaneous activity and responses to 60 g von Frey (Fig. 5 A and 6 A), paw (Figs. 5E and 6E), tail (Fig. 5 F and 6 F) and ear (Fig. 5 G and 6 G) pinch, all intense mechanical noxious stimuli.

#### 3.4.1 Effect of two doses of tapentadol and subsequent administration of naloxone

Tapentadol significantly reduced neuronal responses of the naloxone group to 60 g von Frey (*P*=0.003 after 1 mg/kg and *P*=0.006 after 5 mg/kg; Fig. 5B), paw pinch (*P*=0.029 after 1 mg/kg and *P*=0.016 after 5 mg/kg; Fig. 5E), tail pinch (*P*=0.049 after 5 mg/kg; Fig. 5 F) and ear pinch (*P*=0.007 after 1 mg/kg and *P*<0.001 after 5 mg/kg; Fig. 5 G). Naloxone reverses the effect that tapentadol has in these evoked activity, shown by the lack of significant difference, between baseline and post-naloxone responses to 60 g von Frey (*P*=1.000), tail pinch (*P*=1.000), paw pinch (*P*=0.199) and ear pinch (*P*=0.072).

#### 3.4.2 Effect of two doses of tapentadol and subsequent administration of yohimbine

Tapentadol significantly reduced neuronal responses in the yohimbine group to 4 g von Frey (*P*=0.011 after 1 mg/kg and *P*=0.002 after 5 mg/kg; Fig. 6 A), 60 g von Frey (*P*=0.009 5 mg/kg; Fig. 6B), cold (*P*=0.009 after 1 mg/kg; Fig. 6 C), heat (*P*=0.009 after 1 mg/kg and *P*=0.005 after 5 mg/kg; Fig. 6D), paw pinch (*P*=0.002 after 1 mg/kg and *P*=0.001 after 5 mg/kg; Fig. 6E), tail pinch (*P*=0.013 after 1 mg/kg and *P*=0.012 after 5 mg/kg; Fig. 6 F) and ear pinch (*P*=0.011 after 1 mg/kg and *P*<0.001 after 5 mg/kg; Fig. 6 G). Yohimbine reversed the effect of tapentadol on the responses of CeA neurones to 4 g and 60 g von Frey, heat, tail and ear pinch (*P*=1.000 between baseline and post-yohimbine; Fig. 6 A, B, D, F and G). The reversal was observed on the responses to cold (*P*=0.840 between baseline and post-yohimbine; Fig. 6 C) on the responses to paw pinch (*P*=0.092 between baseline and post-yohimbine; Fig. 6E).

Control studies verified that the effects of the antagonists were not due to the effects of tapentadol wearing off since the effects of 5 mg/kg were maintained for times longer than the antagonist studies.

### 3.5 Full raw recording of a single SNL CeA neurone time matched to the overall population results from Fig 3D

**Fig. 7:**
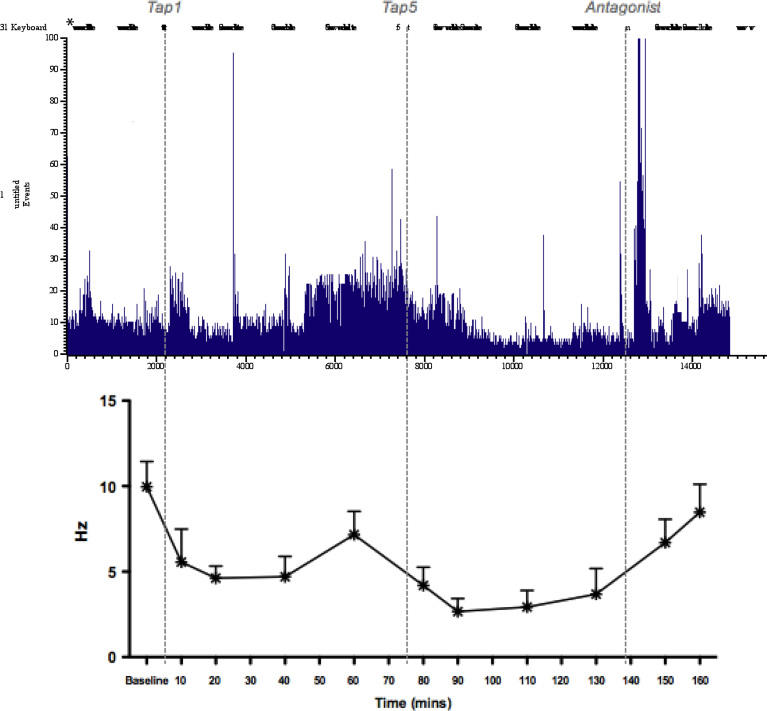
Typical full recording (raw data) of a single SNL CeA neurone and the overall population results from Fig. 1D. Values of firing per second (Hz) are represented on the *y*-axis and time is represented on the *x*-axis. The upper graph represents the raw data, where the black bars at the top represent one test each. Interference is subtracted when the data is extracted. The lower graph shows the effects of the same pharmacological study on the all population of neurones.

As expected, the overall SNL population neuronal activity of (Fig. 7, bottom graph) presents the same pattern of activity of a typical individual SNL neurone (Fig. 7, top graph). Neuronal activity is high at baseline (before *Tap1*), shows a slight decrease after administration of tapentadol 1 mg/kg (*Tap1*) followed by an increase just before the administration of tapentadol 5 mg/kg (*Tap5*), after which neuronal activity becomes decreased in a stronger and more permanent manner. Injection of antagonist (naloxone) results in an increase of neuronal activity to values similar to baseline. The very high and usually isolated lines represent interference, which is subtracted from neuronal activity values in a post-recording analysis.

## 4 Discussion (1543/1500)

Neuroplasticity in the right CeA of animals in pain models (Neugebauer and Li, 2002; Ji and Neugebauer, 2009), including neuropathic pain (Gonçalves and Dickenson, 2012), is likely to play an important role in chronic pain and associated emotional comorbidities (Gonçalves et al., 2008). We found that systemic tapentadol, a MOR-NRI effective in patients with neuropathy, osteoarthritis and low back pain (Games and Hutchison 2013; Kavanagh et al., 2004), reduces spontaneous and evoked activity of right CeA neurones in animals with neuropathy. We also determine that the two components of the inhibitory effects of tapentadol (MOR producing opioid receptor activation and NRI leading to α_2_-adrenoceptor activation) have differential effects on spontaneous and stimuli-evoked activities. The action of tapentadol was entirely dependent on the presence of nerve injury, since there was no inhibitory effect in sham animals, suggestive that the neuronal responses and pharmacological substrates in the amygdala were highly linked to a persistent pathophysiological state.

Both the MOR and NRI components interact with important pain inhibiting systems. Noradrenaline results in acute antinociception in normal and neuropathic pain states through activation of α_2_-adrenoceptors, which decrease neurotransmitter release onto spinal dorsal horn neurones (Eisenach, 1999). Accordingly, the α_2_-adrenoceptor agonist dexmedetomidine inhibits acetylcholine release in spinal cord (Obata et al., 2005). Drugs such as amitryptiline, duloxetine and clonidine are often used to treat chronic neuropathic pain. These drugs either activate, augment, or mimic the descending noradrenergic pathway, stimulating spinal α_2_-adrenoceptors resulting in analgesia (Xu et al., 1992; Xu et al., 1997; De Felice et al., 2011).

Here, the higher dose of tapentadol resulted in inhibition of spontaneous and evoked activity of CeA neurones in SNL animals. Although the effects of tapentadol were statistically significant on the responses to some stimuli only in the yohimbine group (Fig. 6 A, C and D), there is a clear trend also on the naloxone group to lower responses after tapentadol. Sham animals neuronal activity after stimulation was not different from spontaneous activity, as described before (Gonçalves and Dickenson, 2012); this spontaneous activity was also not different before and after tapentadol administration (Fig. 3 C and D), implicating that tapentadol affects CeA neuronal activity only in the presence of neuropathy. While sham animals neuronal activity at baseline is significantly different from SNL animals baseline, after naloxone and yohimbine spontaneous activity is increased to values similar to activity in SNL animals (Fig. 4).

Both components of tapentadol have been implicated in endogenous control of neuropathic pains. After nerve injury the modulating effects of α_2_-adrenoceptors on spinal processing is lost (Rahman et al., 2008). De Felice et al. (2011) reported a marked protective role of noradrenergic systems in controlling the behavioral consequences of SNL neuropathy, and Xu et al. (1992) described a similar role of MORs after spinal cord injury. Shifts in the MOR-NRI components of tapentadol are seen in behavioral tests of mechanical hypersensitivity in nerve injury models where yohimbine was much more effective than naloxone in reversing the analgesic effect of tapentadol (Tzschentke et al., 2007). In SNL and sham animals, atipamezole (α_2_-adrenoceptor antagonist) or naloxone administered spinally almost fully reversed tapentadol effects on all spinal neuronal responses, suggested to be a reflection of the essential synergy between the two components of tapentadol with a shift towards a greater noradrenergic component after nerve injury (Bee et al., 2011). In sham animals, using these spinal sensory neuronal measures, the drug was effective. Here at higher CNS levels, and perhaps related to limbic pain processes, the drug lacked effects in sham animals and the antagonism studies after neuropathy were more complex. Thus, in SNL animals, while naloxone fully reversed 5 mg/kg tapentadol effects on spontaneous activity (Fig. 3 A), and activity evoked by 60 g von Frey and tail pinch (Figs. 2 and 3D), it failed to completely reverse the responses of these neurones to cold, heat and ear pinch and it had no effect on responses to paw pinch. On the other hand, yohimbine administration reversed to baseline values of CeA responses to virtually all stimulation and spontaneous activity to values slightly below to baseline. This indicates that, in the presence of neuropathy, either NRI or MOR components alone can control spontaneous activity and responses to 60 g von Frey and tail pinch. In contrast, synergy between NRI and MOR seems to be essential for the effect of tapentadol on responses to cold, heat and ear pinch. The NRI component alone underlies inhibition of neuronal responses to paw pinch by tapentadol.

Spinally, the same doses of systemic tapentadol reduce mechanical, thermal and electrical-evoked responses of dorsal horn neurones of both SNL and sham rats dose-dependently (Bee et al., 2011). As the doses and time-courses were similar in the brain and spinal cord, the decreased activity observed in the CeA might be consequence of inhibition of ascending activity by tapentadol acting spinally. However, since CeA receives dense ascending noradrenergic innervation (Zardetto-Smith and Gray, 1990; Tully et al., 2007) and has a high density of α_2_-adrenoceptors (Talley et al., 1996) and MORs (Mansour et al., 1995; Poulin et al., 2006), the premise that tapentadol might be acting directly in the AMY through NRI cannot be ruled out.

The same is true for the opioid component. MORs are densely located in the amygdala, playing an important role in reversing mechanical hypersensitivity following nerve injury (Martin et al., 2011). μ-opioid agonist binding in the amygdala and ventral midbrain decreases after chronic nerve injury (Martin et al., 2011; Narita et al., 2006), changes thought to contribute to the establishment and maintenance of chronic pain. Whilst intra-amygdalar administration of opioids results in analgesia through MOR activation (Ozaki et al., 2003), opioid agonists also inhibit the release of noradrenaline in the amygdala (Wilson and Junor, 2008). Whatever the case, the ability of tapentadol to modulate this limbic activity is very different from its spinal actions. In the spinal cord, the MOR and NRI components are different and the absolute dependency on nerve injury for its action is not present.

AMY is highly active during exposure to emotional, high arousing or motivational stimuli (Kawahara et al., 2004; Adolphs et al., 2000; Cahill et al., 1996). Specifically, exposure to different aversive sensory modalities (Fischer et al., 2003; Phan et al., 2004; Zald and Pardo, 2002), including unpleasant interoceptive or painful stimulation consistently activates AMY neurones (Cahill et al., 2000). The AMY has a particular important role in the strengthening and consolidation of long-term emotional memory (Adolphs et al., 2000; Paré, 2003), and noradrenaline is essential to complete this role (McGaugh et al., 1996; McGaugh, 2000; Quirarte et al., 1997). Moreover, systemic or intra-amygdalar administration of opioid receptor agonists and antagonists respectively impair and enhance memory retention (Baker and Kim, 2004; Ferry et al., 1999), indicating that opioids are of functional significance in the regulation of emotional learning and memory (Fanselow and Bolles, 1979), as is noradrenaline (Ferry and McGaugh, 1999; Quirarte et al., 1997). The overlap between the MOR-NRI mechanisms in pain modulation may extend to emotional memory, and this should be looked into in future experiments.

In addition to the synergy between the μ-opioid and α_2_-adrenoceptor components of the actions of the drug, there is also a synergy between the spinal and supraspinal actions of the drug (Schröder et al., 2011). Not only would a single antagonist interfere with this interaction, but the effects of blocking spinal μ-opioid and α_2_-adrenoceptor function spinally may be different at supraspinal sites, as shown. Since tapentadol and the antagonists were given systemically they could act on spinal and brain μ-opioid and α_2_-adrenoceptor systems. Previous studies and our present results suggest that the balance of the opioid and noradrenergic component of tapentadol vary with models and persistence of the pain and CNS sites. The inhibition of noradrenaline release in the amygdala by opioid agonists (Kawahara et al., 2004) might explain why the NRI component is even more predominant at spinal levels where morphine does not alter noradrenaline levels and the ability of tapentadol to elevate noradrenaline levels is not seen in sham animals (Meske et al., 2014). The balance in drugs pharmacological actions will likely vary, depending on the CNS area. This and all the similarities between pain and memory mechanisms lead to the consideration of chronic pain as a disease of CNS plasticity (Price and Ghosh, 2013). The ability of tapentadol to improve quality of life in pain patients may relate to its ability to modulate amygdala activity. Indeed, as we have recently reported in detail (Gonçalves and Dickenson, 2012), there is a time related shift from left to right amygdala after neuropathy in both ongoing and evoked activities of CeA neurones.

## 5 Conclusion

CeA activity of SNL rats is decreased by systemic administration of tapentadol, and this is reversed by systemic naloxone or yohimbine but with differences to the spinal actions of the same doses (Bee et al., 2011). These MOR and the NRI components likely enable tapentadol to be effective with reduced opioid load compared to pure opioids, in that equivalent analgesia can be produced in animals and patients with less side-effects (Riemsma et al., 2011; Schug et al., 1992). In patients, there are also improvements in co-morbidities and quality of life. Thus, tapentadol, as pregabalin (Gonçalves and Dickenson, 2012), is able to suppress spinal sensory neuronal activity and modulate AMY function, the latter likely to be related to the affective components of pain.
